# Dacryocystitis and Its Treatment

**Published:** 1897-02

**Authors:** 


### DACRYOCYSTITIS AND ITS TREATMENT.

In this paper (*The Post Graduate*, November), the writer, Dr. A. E. Davis, confines his remarks especially to the chronic forms of the disease.

In connection with the treatment with styles, Dr. Davis says:

"The latest advocate of this method is Dr. Walter Vulpius, who says he rather welcomes chronic affections of the lachrymal apparatus. He thinks permanent probes are 'indicated in all chronic stenoses of the latchrymal passages with their various sequelæ.'

"In such cases he slits the lower canuliculus, if it has not been done already, and inserts a permanent probe at the first sitting. This probe has a short horizontal part, that lies in the slit canuliculus, while the vertical part extends into the inferior nasal meatus. He says: 'The instrument [probe] had to be adapted to the individual case—this was the chief point—in order to avoid any pressure. The probe was made of common alloyed silver, as used by jewelers, which can be smoothly polished and which is not easily oxidized, so that the surface remains unaltered and non-irritating for an indefinite time. Its thickness corresponds to a Bowman's No. 5. Both ends are blunt, the horizontal a little flattened towards the eyeball.'

"Dr. Vulpius claims that great care has to be exercised in adjusting the horizontal part of the probe; if it turns in too much it presses on the eyeball and irritates that; on the other hand, if it turns outward too far it will avert the lower lid. If the probes are properly adjusted, he claims that they can be worn for an indefinite time without harm; in fact, he has had patients wear them for as long as four years, and with benefit in all cases. He accounts for the drainage of the eye by the solid probes, *in situ*, by a capillary current created between the probe and the wall of the duct."

Dr. Davis gives the following as his conclusions:

"1. In acute cases, irrigation of the sac with antiseptic solutions along with the injection of mild astringents should first be tried.

"2. Slit the canaliculus and pass probes.

"In chronic cases:

"1. If the canaliculus has not already been operated on this should be done at once.

"2. Gradual dilatation of the stricture in the nasal duct by Bowman's probes should follow. The larger the probe that can be passed without traumatism to the duct wall the better. Irrigations and injections may be used in conjunction with probes.

"3. If probing fails, the permanent styles are to be tried.

"4. As a last resort, destruction of the lachrymal sac.

"5. Strict attention should be given to the nose as to examination and treatment in all cases."—*American Medical Review.*

